# Should the Nurse Articulate?

**Published:** 1918-11-23

**Authors:** 


### SHOULD THE NURSE ARTICULATE?

May I suggest this as a subject for debate at College Centres? Since I began to ventilate the economic grievances of the nursing profession in The Hospital I have had many critics, and may I repeat, in passing, what I have I think already stated, that I regard criticism as the most wholesome tonic in the world for the reformer or the propagandist? Without an Opposition Parliament would be an unprogressive institution, and Acts of Parliament often owe their best clauses, not to the men who introduce the Bills, but to those who sit on the other side of the House. I therefore thank my critics, even when they write in wrath.

### The Nurse as a Bondslave.

I think that all those who have followed the arguments carefully will agree with me that they boil down to a single issue—whether it is right and proper for nurses to complain when they have grievances, or whether they should remain silent. With one consent hostile and friendly critics admit that very serious grievances exist. The "London" Hospital has put it on record that nurses are "shamefully underpaid." It is beyond dispute that their hours of labour are much more strenuous and trying than those of any other class of workers, male or female. Housewives are now making active arrangements for permitting domestic servants to live out so that they may have their evenings off duty. The nurse alone among woman workers is a bondslave. I have scores of letters in my mail-bag from nurses begging to have all their meals out of the sick wards, scores of others complaining of the nerve-racking effect of almost never-ceasing duty. Others ask what is to become of them when they are old or infirm, when they are "scrapped" as no longer useful. Here and there somebody suggests that I am not widely informed, and that my correspondents are of the domestic-servant type. But leaving aside such futile and childish arguments, if such they can be called, I think I am correct in stating that there is a consensus of opinion that the economic grievances which I have mentioned actually exist, and the question at issue is, therefore, Should the nurse complain or should she suffer in silence? It is alleged that it is undignified and disloyal to grumble. Lord Knutsford says that a bargain is a bargain, and if the nurse does not like it she should seek other employment. "Liverpool," in last week's Hospital, says "Attack the committees," and also says that good fellowship between committees and the nursing staff is essential. It ought to be obvious that if the nursing staff is all smiles an attack on the committee would only court rebuff.

### The Way to Betterment.

I hold the view, and with profound conviction, that if the nurse means to have her grievances redressed she must press for reform with all the force that she possesses. She must make it unmistakably clear to her employers that her conditions of service are intolerable, and she must insist on betterment. This is as clear to me as that if you want coal to be brought out of the bowels of the earth you must dig for it. It will not come up for whistling! Of course I do not profess infallibility. I merely state my views, which may be erroneous, and if anybody can show me a more excellent way I am open to conviction.

### Methods of Articulation.

Of course there are a variety of methods of articulation. Here, for example, is one. If the College of Nursing were to issue to each of its members a printed form asking them to set out thereon in order of relative importance what in their opinion are the most urgently needed reforms, and then publish the result in the *Times*, and likewise present it to the hospital committees of the kingdom, I have no doubt that excellent results would ensue. As a matter of fact I have been hoping for some such action, and I continue to hope. I understand that it is contemplated to hold a public inquiry into the remuneration question, which is urgent and important. But unless the nurses themselves articulate, and in no uncertain tone, even the advocacy of the committee of the College would be weak. They must have behind them the voice of the members.

### "Bargains" and Efficiency.

And while I am discussing this matter I may add, moreover, that if the College were to take some such action as that which I suggest a very substantial augmentation of its membership would take place, for I am not "sowing seeds of disloyalty, grumblings, and discontent." I am simply voicing feelings that already exist and that are driven beneath the surface by advocates of loyalty falsely so called. It is notorious that the nurse is scandalously overworked, scandalously underpaid; it is notorious that hospital matrons are afraid to press these facts on their committees, and it is likewise notorious that these conditions of employment—these "bargains"—interfere with the efficient nursing of the nation's sick and drive excellent would-be candidates for nursing into other employments.

### A National Question.

This is a national question. Hospital committees are public bodies, and must be approached as such. The College of Nursing is the nurses' representative body, and owes an immediate and pressing duty to its members, and no possible good can accrue to it by an indefinite postponement of what is an urgent necessity. I am, of course, aware that the committee of the College consists almost entirely of matrons in active service, and that for them to "attack the committees," as "Liverpool" puts it, may be a delicate function. But, on the other hand, it is for such delicate functions that they are there. The emancipation of the nurse depends on what I may call the faithful discharge of their Parliamentary duties.

I am quite serious when I state that the subject "Should the nurse articulate?" ought to be debated and talked about in public and private by all whom it concerns. It is either right or wrong, necessary or unnecessary, and matron and junior nurse should alike arrive at a reasonable and logical conclusion. Personally I am free from doubt. I have never yet heard of reform without agitation, and where large bodies are concerned such agitation must be the joint work of all concerned. You know my argument about the walls of Jericho which fell when all the people shouted. Since that historic incident millions of Jerichos have fallen, but I have never heard of one that succumbed to a policy of a smiling countenance and a silent tongue As Mr. Gladstone put it—"Agitate! agitate!! agitate!!!"

